# Arabinoxylan-Carboxymethylcellulose Composite Films for Antibiotic Delivery to Infected Wounds

**DOI:** 10.3390/polym14091769

**Published:** 2022-04-27

**Authors:** Nabil K. Alruwaili, Naveed Ahmad, Abdulaziz I. Alzarea, Fadhel A. Alomar, Ali Alquraini, Sultan Akhtar, Muhammad Syafiq Bin Shahari, Ameeduzzafar Zafar, Mohammed Elmowafy, Mohammed H. Elkomy, Anton V. Dolzhenko, Mohammad Saeed Iqbal

**Affiliations:** 1 Department of Pharmaceutics, College of Pharmacy, Jouf University, Sakaka 72388, Saudi Arabia; nkalruwaili@ju.edu.sa (N.K.A.); azafar@ju.edu.sa (A.Z.); melmowafy@ju.edu.sa (M.E.); mhalkomy@ju.edu.sa (M.H.E.); 2 Department of Clinical Pharmacy, College of Pharmacy, Jouf University, Sakaka 72388, Saudi Arabia; aizarea@ju.edu.sa; 3 Department of Pharmacology, College of Clinical Pharmacy, Imam Abdulrahman Bin Faisal University, Dammam 31441, Saudi Arabia; falomar@iau.edu.sa; 4 Department of Pharmaceutical Chemistry, Faculty of Clinical Pharmacy, Al Baha University, Al Baha 65779, Saudi Arabia; aalquraini@bu.edu.sa; 5 Department of Biophysics Research, Institute for Research and Medical Consultations, Imam Abdulrahman Bin Faisal University, Dammam 31441, Saudi Arabia; suakhtar@iau.edu.sa; 6 School of Pharmacy, Monash University Malaysia, Jalan Lagoon Selatan, Bandar Sunway 47500, Malaysia; muhammad.binshahari@monash.edu (M.S.B.S.); anton.dolzhenko@monash.edu (A.V.D.); 7 Department of Chemistry, Forman Christian College, Ferozepur Road, Lahore 54600, Pakistan; saeediqbal@fccollege.edu.pk

**Keywords:** film dressing, antibiotic delivery, arabinoxylan, carboxymethylcellulose, infected wound

## Abstract

Modern dressings should provide for local delivery of antibiotics and protect the wound from bacterial infection, dehydration and environmental factors to achieve optimal healing. The local delivery of antibiotics can reduce adverse effects and resistance challenges. In this study, we fabricated film dressings composed of arabinoxylan (AX) from Plantago ovata seed husks and carboxymethylcellulose (CMC) by a solvent cast method for the delivery of the antibiotic amikacin (AMK). To determine the suitability of the prepared AX-CMC composite films as wound dressings and drug delivery materials, their physical, chemical, mechanical, morphological, thermal, pharmaceutical, antimicrobial, cytocompatible, and drug delivery properties were investigated. The results demonstrated that the dressings were suitable for delivering the drug at the wound site in a sustained manner and keeping the environment moist for rapid healing. The AMK-loaded AX-CMC films exhibited controlled release of AMK, excellent antibacterial activity, and cytocompatibility. Thus, the AX-CMC composite films appear to be promising bioactive dressing materials for the prevention of wound infections.

## 1. Introduction

Bacterial infection is one of the major challenges that can prolong wound healing and lead to chronic wounds [1,2]. Topical antibiotics containing dosage forms are frequently prescribed for application to the wound site with the aid of dry wound dressings to prevent or reduce infection [3]. Dry dressings consist of materials such as cotton, textile gauze, and pads to absorb wound exudates, which may result in desiccation of the wound and sticking of the dressing to the skin [3,4,5]. Moreover, the peeling of such dressings may cause pain and trauma, resulting in poor comfort and making the wound more prone to infection [3]. Additionally, the dry state of the wound prolongs healing by slowing the migration of cells. Therefore, a moist environment is considered conducive to rapid/optimal wound healing, which has led researchers to develop moist dressing materials such as films, foams, and hydrogels [4,6]. In addition, moist dressing materials can be used to deliver different types of drugs at controlled rates to prevent infections and augment the healing process [6,7,8]. In recent decades, enormous attention has been focused on developing biocompatible dressing materials that can maintain a moist environment, deliver antibiotics, absorb wound fluid, permeate water vapors, and promote epithelization [8,9,10].

Among these dressings, polymeric films are a popular choice because of their ease of preparation, application, water absorption, and biodegradable characteristics [11,12]. For this, numerous swellable natural, semisynthetic and synthetic polymers are being explored to develop efficient wound dressing materials [8,13,14]. The challenges faced in developing these materials include the poor mechanical strength and biocompatibility of the material and controlled release of the drug [9,12,15]. Therefore, efforts are directed to identify polymers of natural origin from renewable sources and prepare combinations of these polymers to overcome these challenges [9,16].

Arabinoxylan (AX) from *Plantago ovata* seed husk presents water-swellable, biodegradable, film-forming, and biocompatible properties and thus is an attractive polymer for application in wound dressings and drug delivery [12,13]. Previously, gentamicin–loaded AX-based films exhibited promising potential for wound dressing application, but there was a need to improve the integrity of the films in a simulated wound environment and prolong the drug release [13]. Combinations of different biopolymers equip dressing materials with the advantages of controlled drug delivery and wound healing [4]. Therefore, in subsequent studies, films were prepared by combing AX with gelatin (GL) and sodium alginate (SA) that resulting in the improvement in the integrity of the films in a simulated wound environment [12,17]. However, AX–GL and AXSA film’s flexibility was lesser than AX films [12,13,17]. Moreover, expansion of the AXSA films was lesser than AX films, resulting in lower drug release [17].

Carboxymethylcellulose (CMC), a derivative of cellulose (most abundant biopolymer), is an approved excipient in formulations of various conventional and advanced dosage forms in the pharmaceutical industry [9,18], and it is a widely explored material for wound dressings [5] because of its hydrophilicity, biocompatibility, low immunogenicity, and ability to increase cytokines [5,19]. However, the ionization of carboxylic groups present in the chemical structure of CMC makes it highly sensitive to water absorption, which may lead to the poor mechanical integrity of the films in a wounds environment and rapid drug release [19,20]. One of the strategies to improve the physiochemical properties of polymeric films is the preparation of composite films. Previous studies demonstrated that combining different polysaccharides in single films formulation improves the films’ physical, mechanical, drug release, and wound healing properties [5,21,22,23]. Therefore, AX was combined with CMC in this work. It was hypothesized that the hydrogen bond interaction between the hydroxyl and carboxylic groups of AX and CMC could result in improvement in the mechanical properties of the films. Moreover, the involvement of carboxylate groups of CMC in hydrogen bonding could overcome the rapid water absorption and rapid drug release associated with it. To the best of our knowledge, the present study is the first report on the combination of AX and CMC in a film formulation.

Amikacin (AMK) is a second-generation (synthetic) aminoglycoside that exhibits a broad-spectrum bactericidal effect by binding with the 30S ribosomal subunit and interfering with ribosomal protein synthesis [24]. Conventionally, AMK is administered through a parenteral route (IM or IV) that is associated with an abrupt increase in blood concentration and requires monitoring of its blood level [24,25]. Moreover, systematic AMK therapy is also associated with adverse effects (ototoxicity, nephrotoxicity) and the development of AMK resistance [24,25]. Therefore, local (topical) delivery of AMK can be employed to overcome these problems [25]. Previously, AMK-loaded dressing (sponges, injectable gels, and hydrogels) systems have demonstrated promising results of their local application in wound healing [25,26,27,28]. Therefore, AMK is selected as a model antibiotic for loading and delivery using AX- and CMC-based films.

Thus, the objective of this work was to fabricate, optimize and characterize AX-CMC composite films for the local delivery of AMK to infected wounds. The AX-CMC composite films were prepared by the solvent casting method, and their potential as a wound dressing and drug delivery system was evaluated by physicochemical, pharmaceutical, antibacterial, and cytocompatible analyses.

## 2. Materials and Methods

### 2.1. Materials

CMC, glycerol (GLY), AMK, NaOH, HCl, penicillin, streptomycin, Mueller–Hinton (MH) agar, 3-(4,5-dimethylthiazol-2-yl)-2,5-diphenyltetrazolium bromide (MTT) solution, PBS tablets, silica gel beads, and transdermal diffusion membranes were obtained from Millipore Sigma (St. Louis, MO, US) and used without any modification. The human fetal lung fibroblast cells (MRC-5) (ATCC CCL-171) and bacterial strains (*Escherichia coli* (ATCC 8739), *Pseudomonas aeruginosa* (ATCC 9027), and *Staphylococcus aureus* (ATCC 25923)) were procured from the American Type Culture Collection (ATCC, Manassas, VI, USA). The culture medium RPM1-1640 and fetal bovine serum (FBS) were obtained from Nacalai Tesque (Kyoto, Japan) and Biosera (Nuaille, France), respectively. All experiments were performed in double-distilled water (DDW) unless otherwise specified. AX was extracted and characterized from *Plantago ovata*/psyllium seed husk (PSH) using our previously described method [12,29].

### 2.2. Preparation of AMK-Loaded AX-CMC Composite Films

The AX-CMC composite films were fabricated by using the solvent casting technique. For this purpose, AX, CMC, and GLY were added to DDW to obtain aqueous gels of different compositions (Table 1) by continuous stirring at 500 rpm for 5 h. The gels were sonicated for 30 min using a sonicating water bath to remove entrapped air. Thereafter, to cast blank AX-CMC composite films, the gel (25 g) was poured into Petri dishes (95 mm diameter) and dried at 35 °C for two days in an oven. The physical appearance of the resulting AX-CMC films was assessed to select the optimum film casting composition. The AMK-loaded AX-CMC films were prepared as above by mixing the AMK aqueous solution (0.2% *w*/*w*) with an AX-CMC gel. The compositions of the prepared AX-CMC composite films are given in Table 1. After drying, the blank and AMK-loaded films were peeled off from the Petri plates and stored in a desiccator until further analysis.

### 2.3. Physiochemical Characterization of the AX-CMC Films

The thicknesses of the AX-CMC composite films were determined at five different areas using a screw gauge (APT, Nebraska, USA). The water evaporated from the AX-CMC gels during the drying process was determined using a previously described equation [13].

The water vapor permeation rate (WVPR) of the AX-CMC composite films was studied by adapting a previously reported procedure [15]. The AX-CMC composite films were fastened on the opening of glass vials containing desiccant (silica gel beads dried at 105 °C) and covered with a cap with a one cm^2^ hole. The vials were weighed and maintained in a desiccator at 25 ± 2 °C and 85 ± 5% RH. Thereafter, the weights of the vials were recorded at predetermined time intervals up to 24 h, and the WVPR was calculated.

The tensile strength (TS) and percent elongation at break (% EB) of AX-CMC composite films were determined according to ASTM D882-18 [30] using a universal testing machine (UTM) (LS5, Lloyd Instruments, SXW, UK). The films were cut into dog-bone shapes (5 mm width and 33 mm length) and stretched at a rate of 10 mm min^−1^ until broken. The TS and % EB were calculated as described previously [10].

The differences in the morphologies of the AMK-loaded and blank AX-CMC composite films were observed with scanning electron microscopy (SEM) (Inspect S50, FEI, Brno, Czech Republic). Briefly, the AX-CMC composite films were gold-coated (Quorum, Q150 R ES, UK) and their images were acquired at 20 keV.

Fourier transform infrared (FT-IR) analyses of the pure components and AX-CMC composite films were performed to analyze the composition and chemical compatibility of the films with the drug. An FT-IR-7600 spectrometer (Lambda Scientific, Magill, Australia) coupled with an ATR assembly (PIKE, Madison, WI, USA) was used. The spectra were scanned 64 times in range from 4000 to 550 cm^−1^ at a resolution of 4 cm^−1^.

The thermal behavior of the AX-CMC composite films and their components was studied by thermogravimetric analysis (TGA) and differential scanning calorimetry (DSC). TGA was performed by a TGA-50 Thermogravimetric Analyzer (Shimadzu, Kyoto, Japan). For this, the sample (~20 mg) was heated from ~30 to 600 °C at a rate of 20 °C min^−1^ under N_2_ flow (20 mL min^−1^). The loss in the weight of the sample as a function of time was recorded. DSC analysis of the films and their components was performed using a DSC3 STAR calorimeter (Mettler Toledo, Columbus, OH, US). The sample (~15 mg) was heated from ~30 to 500 °C at a rate of 20 °C min^−1^ under N_2_ flow. The plots of heat flow (mW) vs. temperature were obtained.

X-ray diffraction (XRD) analysis of the selected AX-CMC composite films and their components was performed using a MAXima_X XRD-7000 diffractometer (Schumadzu, Kyoto, Japan) with Cu (Kα) radiation. The diffractograms of the samples (~2 g) were obtained over a 2*θ* range of 5 to 80° at a current of 30 mA, voltage of 40 keV, and scanning rate of 2.0 deg.min^−1^.

### 2.4. Pharmaceutical Characterization of AMK-Loaded AX-CMC Composite Films

The expansion (%) capacity of the AX-CMC composite films was investigated by measuring the increase in the diameter of the film in gelatin solution (4% *w*/*v*, simulated wound environment), according to reported method [21]. Briefly, the gelatin solution (~20 mL) was poured into plastic plates and left for 16 h at 25 °C to solidify as gel. The films (diameter ~25 mm) were kept over the gelatin solution, the increase in diameter was recorded at predetermined time intervals, and the swelling/expansion percentages were calculated using a previously described equation [13].

AMK release from the films was investigated using an automated vertical diffusion cell apparatus (DHC-6T, Logan-Instruments, Somerset, NJ, USA). The release was studied at 37 °C using PBS (pH 7.4) as the release medium in the receiver compartment. Artificial transdermal diffusion membranes (cellulose acetate) were mounted on the top receiver compartments, and the AMK-loaded films were mounted on the artificial membranes. The release medium was automatically sampled from the receiver compartment at programmed time intervals. The amount of AMK released from the films was determined using a Genesys 10S UV–Vis spectrophotometer (Thermo Fisher Scientific, Waltham, MA, USA) as per the reported method [31]. The mathematical kinetic release models (zero-order, first-order, Higuchi and Korsmeyer-Peppas equations) were fitted to release data to determine the release kinetics of AMK from AX-CMC composite films.

### 2.5. Antimicrobial Activity of AMK-Loaded AX-CMC Composite Films

The antibacterial efficacy of the AMK-loaded AX-CMC composite films was determined against *E. coli*, *P. aeruginosa*, and *S. aureus* by the disk diffusion method (~7 mm diameter). AMK (0.2% *w*/*v*, 50 µL) solution (positive), blank disks (negative), and blank composite films (blank) were used as controls. Standardized inocula (20 µL) of the strains under investigation were uniformly spread over M-H agar (20 mL) solidified in glass plates. Sterile test sample disks were placed on the bacterial strains and incubated at 37 °C for 24 h. Thereafter, clear inhibition zones formed around the samples were measured to estimate the antibacterial effect of the drug-loaded films.

### 2.6. Cell Viability of AMK-Loaded AX-CMC Composite Films

MRC-5 (normal lung fibroblasts)cells were cultured in RPM1-1640 medium containing 10% *v*/*v* FBS and 1% *v*/*v* penicillin/streptomycin solution and incubated at 37 °C in a humidified environment in a 5% CO_2_ incubator (Thermo Fisher, Waltham, MA, USA). Then, ~7500 cells per well were seeded in a 96-well plate (NEST, Wuxi, Jiangsu, China) and incubated for 24 h. Thereafter, extracts of selected AX-CMC composite films, prepared by a previously reported method [15], were added to the treatment wells and incubated for 48 h. The percent cell viability (% CV) was determined by performing an MTT assay as previously reported [32]. The absorbances of the plates were recorded at 570 nm using a microplate reader (Molecular Devices SpectraMax iD3, San Jose, CA, USA), and the % CV was determined by comparing the absorbance of AX-CMC film-treated cells with that of untreated cells (vehicle control) using the following equation:

(1) $$\%\;\mathrm{CV}=\left[\;\frac{\mathrm{Absorbance}\;\mathrm{of}\;\mathrm{treated}\;\mathrm{cells}}{\mathrm{Absorbance}\;\mathrm{of}\;\mathrm{untreated}\;\mathrm{cells}}\;\right]\times \;100$$

### 2.7. Statistical Analysis

Experiments were performed in three replicates, and statistically significant differences (*p* < 0.05) were calculated by one-way analysis of variance (ANOVA), Tukey’s test, and nonparametric *t* test using GraphPad Prism V 5.02^®^ software.

## 3. Results and Discussion

### 3.1. Preparation of AMK-Loaded AX-CMC Composite Films

The dried AX-CMC composite films obtained by the solvent cast method were physically evaluated to find the optimum concentrations of AX, CMC, and GLY required for casting films suitable for wound dressing. For this purpose, the obtained films’ peelability, foldability, and smoothness were assessed. The peelability indicates ease of removing films from the plate, and smoothness confirms uniform mixing of the components, while the foldability suggests the flexibility of the films. The physical assessment results of the AX-CMC composite films are presented in Table 1. As a result of a preliminary study, the AXC1 films were rejected due to their rigidity, which might be attributed to the presence of a lower quantity of plasticizer (1% GLY). Therefore, the GLY concentration was increased to 2% in the subsequent formulation, resulting in an improvement in flexibility. Consequently, films coded as AXC2, AXC3, and AXC4 were found to be suitable for application as wound dressing materials. However, AXC5 contained a lower concentration of AX and CMC than AXC2, AXC3, and AXC4 and resulted in very thin films that were difficult to remove from the mold and wrinkled upon removal. Attempts were also made to cast AX-CMC films using higher concentrations (>2%) of AX and CMC, which produced casting gels that were too thick to homogenize and pour. Therefore, the compositions AXC2, AXC3, and AXC4 were selected to prepare AMK-loaded films, and they were coded as AXC2A, AXC3A, and AXC4A, respectively. The resulting AMK-loaded AX-CMC composite films exhibited physical characteristics similar to those of the corresponding blank films. Optical images of the AX-CMC films are shown in Figures S1 and S2. These images suggest that both the blank and the AMK-loaded films were transparent. The transparency of the dressing materials provides medical professionals with the advantage of viewing the progress of healing [11].

### 3.2. Physiochemical Characterization of AMK-Loaded AX-CMC Films

The thicknesses of all the blank AX-CMC composite films (Table 2) were found to be significantly similar. Moreover, the thicknesses of all the AMK-loaded films were also comparable. However, films containing 2% CMC exhibited slightly higher thicknesses. On the other hand, the thicknesses of the AMK-loaded films were greater than those of the blanks. This difference can be understood based on previously reported information on the influence of the flatness of the plates and drying surface, the amount of solids in the formulation, the nature of the polymer, and the casting gel volume on the thickness of the polymeric films [33]. Since AX-CMC composite films differ in the composition and amount of polymer, a difference in the film thickness was expected. However, the surfaces of the films were smooth, and the thickness at various points of the same film was consistent.

The water loss (%) during drying of all AX-CMC composite films did not significantly differ (Table 2). It was observed that the weights of the composite films were higher than the collective weights of the formulation components; this suggests that residual moisture remained there in the films even after drying.

The suitability of polymeric films as wound dressings depends on the WVPR of the films. A good film dressing should not have a WVPR value that is too high or too low because a high WVPR can result in adhesion of the dressing to the wound, and a low WVPR may cause tissue maceration [10,34]. The results of the WVPR of the AX-CMC composite films (Table 2) indicate that the WVPR of AXC2 and AXC2A was lower than that of the other AX-CMC composite films. The primary factors that influence the WVPR include thickness, chemical composition, and porosity. As described above, the AX-CMC composite films were smooth and uniform in thickness; therefore, the lower WVPR of AXC2 and AXC2A can be attributed to a higher solid content than other AX-CMC films. The The WVPR of AX-CMC films are higher than previously reported AX and CMC-based films [12,17,35]. The reported WVPR value of normal skin is approximately 200 g m^−2^ day^−1^ [33], and it may reach 4200 g m^−2^ day^−1^ in injured skin. Thus, wound dressings with WVPR values in the range of 100–3300 g m^−2^ day^−1^ are available on the market [15,33]. Hence, the WVPR values achieved in the present work indicate that the AX-CMC films are suitable for wound dressing applications.

The mechanical properties (TS and EB %) are among the most important criteria to assess the suitability of the polymeric films for wound dressing applications. The dressing material should have sufficient TS to bear the pressure during the application and be flexible for application at moving parts of the body (joints, knees, elbow etc.) [17]. Therefore, the film dressing materials with mechanical strength in the range of human skin (2.5–16 MPa) are considered suitable for wound dressing application [13,36]. The results of the mechanical tests (Table 2) indicate that the TS of AX-CMC composite films ranged between 2.5 to 3.5 MPa while % EB ranged between 90 to 120 %. The TS AX-CMC films containing 1.5% CMC (AXC3 and AXC3A) films were lesser than films containing 2% CMC (AXC2, AXC4, AXC2A, and AXC4A) which might be attributed to stronger hydrogen bonding between the hydroxyl groups of AX, carboxylic groups of CMC, and amide groups of AMK at higher CMC concentration in the AX-CMC films [17,37]. Moreover, the TS of the AMK-loaded AX-CMC films was higher than blank films, which might be due to the higher thickness due to the incorporation of the AMK which may form intermolecular hydrogen bonds with AX and CMC. These results suggested that the combination of the AX with CMC improved the tensile strength and flexibility of the AX-based films [12,13,17]. The TS AX-CMC films were within the range of the normal skin, indicating the suitability of the films for wound dressing application [36].

**Figure 1:**
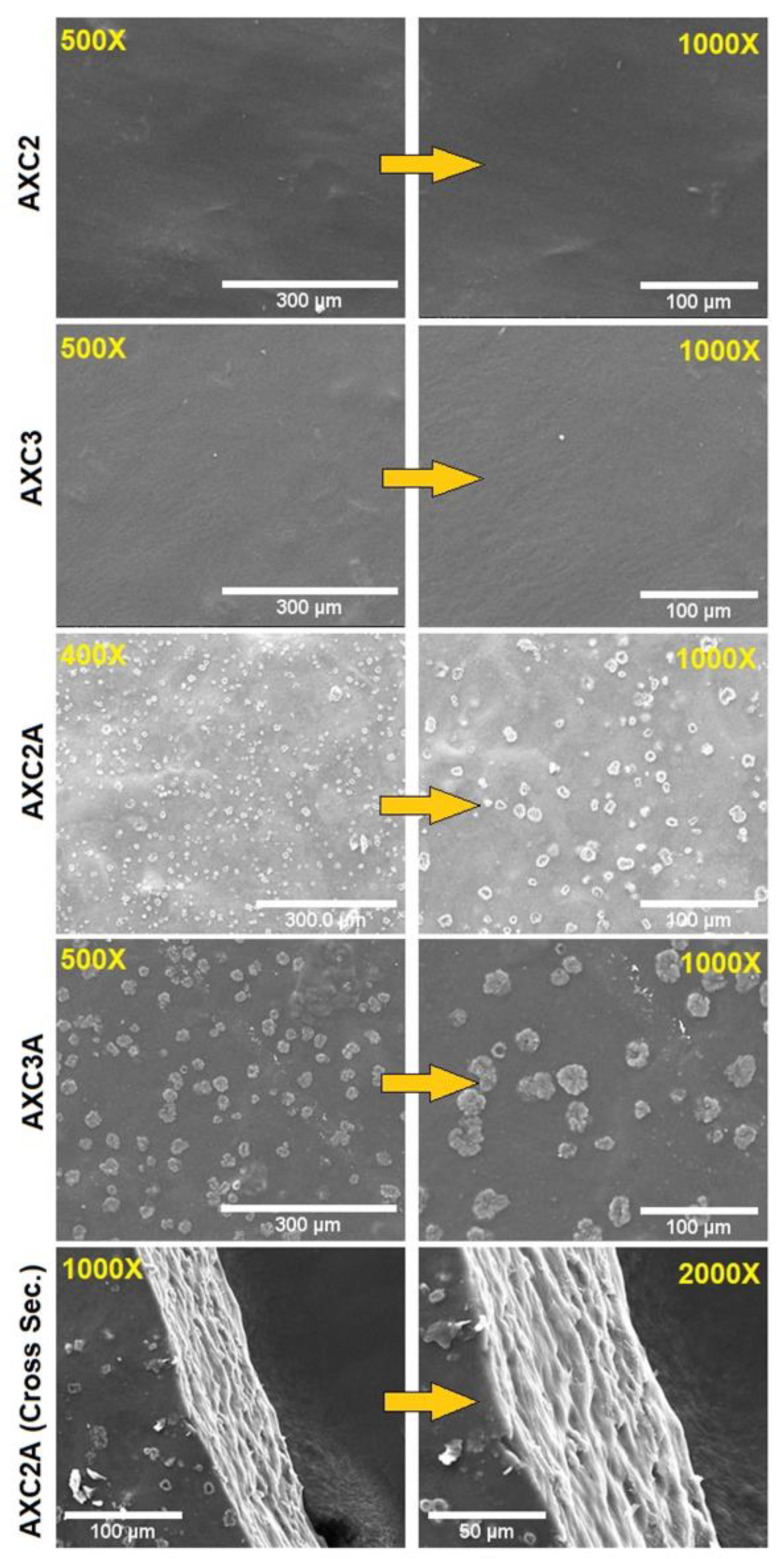
SEM images of the AX-CMC composite films.

The surface and cross-sectional morphologies of the selected blank and AMK-loaded AX-CMC composite films are presented in Figure 1. The blank films (AXC2 and AXC3) exhibited a continuous plain surface morphology. In the case of AMK-loaded films (AXC2A and AXC3A), the particles were found that might be AMK particles homogeneously dispersed throughout the surface of the films [17]. Moreover, the surface morphologies of the AX-CMC composite films were free from any air bubbles and fissures, which indicates homogeneous mixing of AX and CMC by the procedure used [38]. The SEM image of the cross-section of AXC2A further supports the high homogeneity of the blend and lack of pores in the films. These results align with numerous studies [6,38,39,40] of polymeric films intended for wound dressing applications.

**Figure 2:**
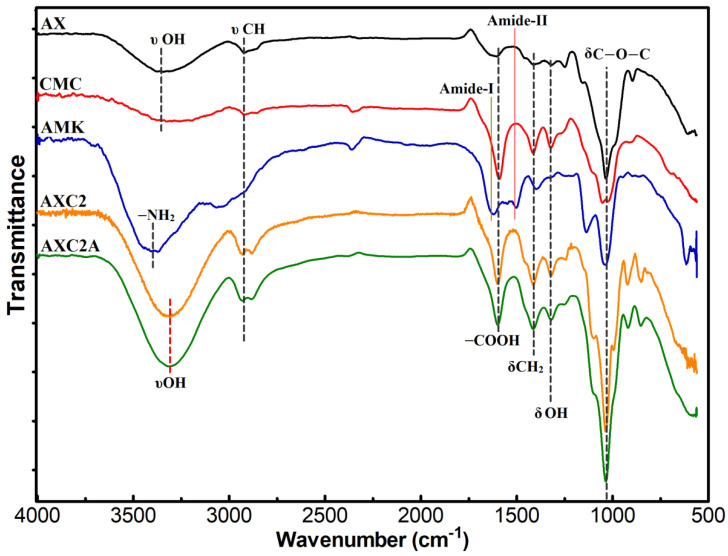
FTIR spectra of the AX, CMC, AMK, AXC2 (blank), and AXC2A (loaded) films.

FTIR-ATR analyses were performed to investigate the possible chemical interactions among AX, AMC, GLY, and AMK during AX-CMC film preparation. The FTIR spectra of AX, CMC, AMK, GLY, AXC2 (blank AX-CMC film), and AX2CA are presented in Figure 2. The spectrum of AX presents absorbance due to –OH, –CH, –COOH groups and glycosidic linkages at approximately 3375, 2925, 1610, and 925 cm^−1^, respectively. This spectrum is consistent with the previously extracted AX from PSH [41]. The signature peaks of CMC were observed in its spectrum at 3375 (–OH), 2925 (–CH), 1605 (–COO^–^), 1425 (–CH_2_), 1335 (–OH bending) 1060 (C–O–C) cm^−1^ [42]. Similarly, the spectrum of AMK exhibited characteristic peaks at approximately 3438, 1643, 1540, 1421, 1210, and 1054 cm^−1^ corresponding to N–H, primary and secondary amide, carboxylate of amino acid, C–H, and C–O–C vibrations, respectively [43].

The signature peaks of CMC and AX were found in the FTIR spectra of AXC2 and AXC2A; however, the hydroxyl group peaks were observed at lower wavenumbers (~3350 cm^−1^) than those in AX and CMC (~3375 cm^−1^), which presented higher intensities. This shift may be attributed to intermolecular hydrogen bonding between film components [37]. The peaks due to AMK in AXC2A could not be observed because of the high dilution effect caused by uniform dispersion of drug particles in the polymer matrix. The absence of any new peak in the spectra of the blank and AMK-loaded AX-CMC composite indicates that the components of the films do not react chemically during the preparation process [12].

**Figure 3:**
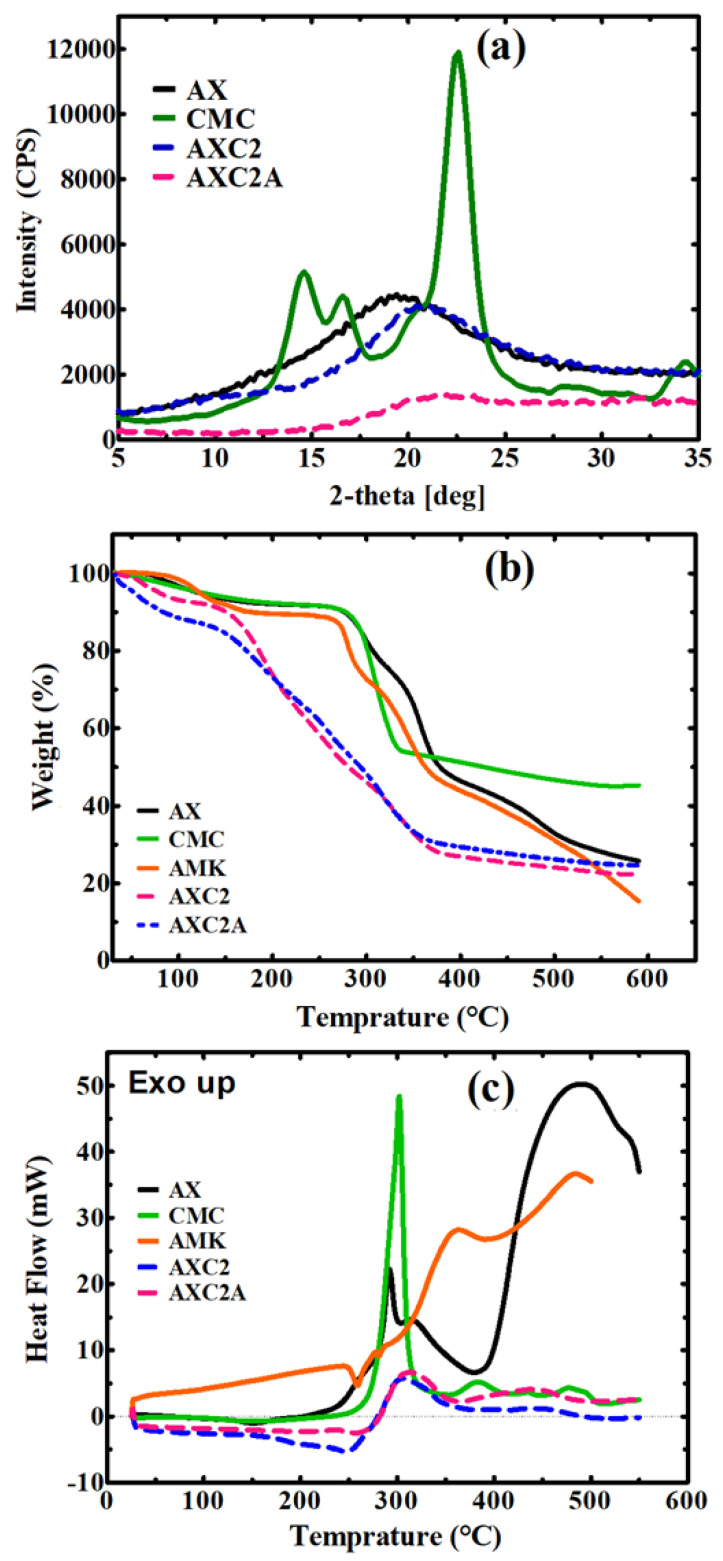
(**a**) XRD, (**b**) TGA and (**c**) DSC analyses of the AX-CMC films.

XRD analysis of AX, CMC, AXC2, and AXC2A was performed to determine the crystallinity of the films. The diffractograms of the films are shown in Figure 3a. The XRD spectrum of CMC exhibited characteristic peaks at 14.6°, 16.6° ((1 0 1) cellulose-I), 20.0° ((1 1 0) cellulose-II) and 22.6° ((0 0 2) cellulose-1) [44]. The crystalline structure of cellulose is attributed to strong intramolecular hydrogen bonding. The spectrum of AX did not exhibit any sharp peak due to its amorphous nature [45]. Similarly, no sharp peak was observed in the spectra of AXC2 and AXC2A films, suggesting that crystalline CMC was converted to amorphous material in AX-CMC films due to the weakening of intramolecular hydrogen bonding in CMC upon complexation with AX, GLY and AMK [42]. Saadiah et al., 2019 reported a similar decrease in CMC crystallinity while preparing CMC/PVA blended films [42].

The thermal degradation behaviors of AX, CMC, GLY, AMK, AXC2, and AXC2A were investigated by TGA. The TGA curves are presented in Figure 3b and Figure S3, while details of the major degradation steps are described in Table S1. AX decomposed in three steps at ambient temperature −125 °C (∆W = 5.27%), 251–384 °C (∆W = 42.7%), and >385 °C (∆W = 22.2%), which corresponded to loss of moisture, fragmentation of AX and complete pyrolysis of AX, respectively. The decomposition behavior of AX is consistent with previous findings [13].

Thermal degradation of pure CMC also occurred in three steps at ambient temperature −125 °C (∆W = 5.06%), 256–335 °C (∆W = 37.2%), and >335 °C (∆W = 9.6%), and these weight losses were attributed to evaporation of moisture, depolymerization, and pyrolysis of the cellulose backbone, respectively [46]. AMK also exhibited three-step degradation at ambient temperature −130 °C (∆W = 5.06%), 254–367 °C (∆W = 40.2%), and >370 °C (∆W = 30.9%), while GLY underwent single-step decomposition at 125–280 °C (∆W = 99.53%) [13,47]. Although the AXC2 and AXC2A films also exhibited three-step degradation similar to pure components, four major differences were observed in the TGA curves of these films: (i) moisture evaporation at higher temperatures, (ii) lower onset temperature of the second step, (iii) longer second step with a high %∆W and (iv) lower residual weight. The early onset and lower residue in the case of AXC2 and ACX2 can be attributed to the presence of GLY, while the longer second step can be due to overlapping of the individual degradation steps of the components of the AX-CMC films. The residual weight of the AMK-loaded film (AXC2A) was higher than that of the blank film (AXC2) due to the presence of ash content in the drug [11,13].

The results of the DSC analysis are depicted in Figure 3c. The DSC curve of pure CMC exhibited a sharp decomposition exotherm at ~301 °C [48] that corresponds with the second step of TGA. The curve of AMK showed a melting endotherm at ~255 °C and a broad decomposition exotherm at ~352 °C [49]. Moreover, the DSC curves of pure AX and GLY are similar to previously reported results [12]. Unlike CMC, AX, and GLY, sharp endo- or exotherms were not observed in the case of the AXC2 and AXC2A films. In the blank film (AXC2), a broad endotherm (like GLY) and decomposition exotherms were observed at ~263 °C and ~307 °C, respectively. The AMK-loaded film (AXC2A) exhibited a deeper endotherm than AXC2 due to the melting of AMK. The presence of the melting peak of AMK in the composites indicated that the drug did not chemically interact with the other components. Moreover, in AXC2 and AXC2A, the decomposition exotherm of AX was shifted to a lower temperature due to the presence of CMC and GLY. These findings are in line with previous studies [12,13]. The thermal study indicates that the composites are stable up to ~250 °C.

### 3.3. Pharmaceutical Characterization

**Figure 4:**
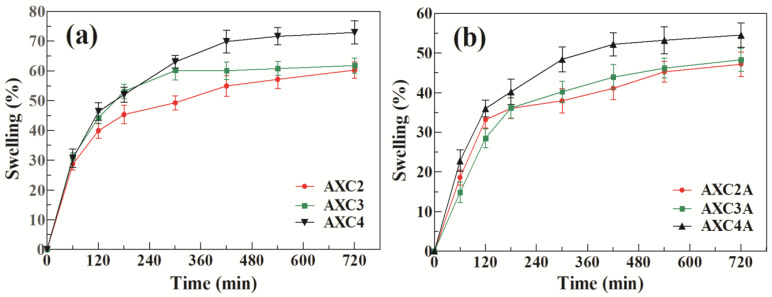
Expansion profiles of the (**a**) blank and (**b**) AMK-loaded AX-CMC composite films.

The results of the expansion studies of the blank and AMK-loaded AX-CMC composite films are presented in Figure 4. The expansion studies were performed in a 4% gelatin solution reported to solidify and produce a moist surface, such as for exudating wounds [21,50,51]. The results of the expansion studies suggest that blank AX-CMC composite films swelled and expanded up to ~70% in 12 h, with the highest expansion percent for AXC4, followed by AXC3 and AXC2. Similarly, among AMK-loaded films, AXC4A exhibited the highest expansion after 12 h, followed by AXC3A and AXC2A. The higher expansion of AXC4 and AXC4A can be explained by the greater amount of CMC in these AX-CMC films, which ionize and undergo more expansion due to electrostatic repulsion among the carboxylate ions [52]. Moreover, the expansion rate of both the blank and AMK-loaded AX-CMC films was rapid in the first 3 h, slowed down gradually, and became minimal after 8 h. Optical images of AXC2 and AXC2A (Figure S4) show that the AX-CMC films retained their shape and did not convert to free-flowing gel during expansion studies. This expansion behavior of AX-CMC composite films was better than previously reported AX and AX-GL films, which lost their integrity [12,13,17]. Overall, expansion of the AMK-containing films was less than that of the blank AX-CMC films due to the higher TS of the AMK-loaded films (due to polymer drug hydrogen bonding interaction). A similar decrease in the swelling/expansion behavior of CMC and AX-based films after drug loading has been reported in previous studies [12,52].

Swellable wound dressing materials augment the wound healing process by absorbing wound exudates and maintaining a moist environment that helps in cell migration and differentiation [9,52]. Moreover, swelling also allows control of the release of a drug from the delivery system [11]. The expansion characteristics of the AMK-loaded AX-CMC composite films indicate that these films can promote exudate absorption and cell migration and deliver drugs in a sustained manner at the wound site.

**Figure 5:**
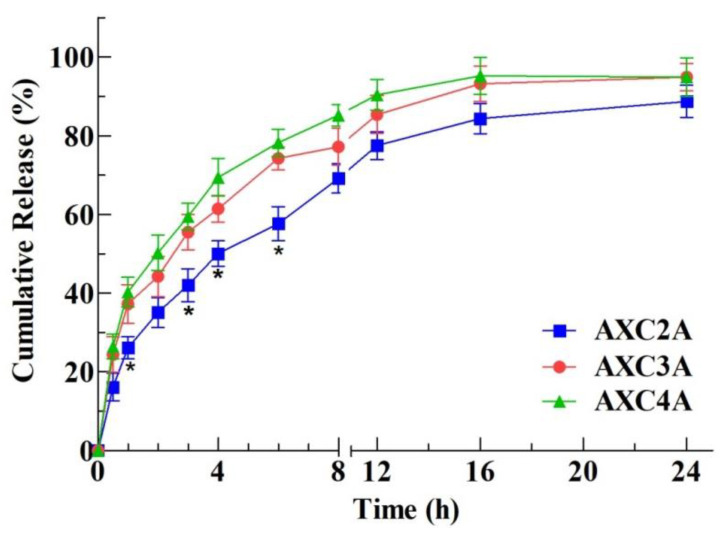
Cumulative AMK release from AMK-loaded AX-CMC films (mean ± SD, *n* = 3, * *p* < 0.05).

The results of the AMK release from the drug-loaded AX-CMC films under investigation are depicted in Figure 5. The results demonstrate that all tested AX-CMC films exhibited a sustained release of AMK and released >90% AMK over 24 h. The drug release from all the films was faster in the first 4 h, possibly due to the dissolution of the adsorbed AMK on the surface of films [11]. However, the AMK release from AXC2A film was significantly lesser than AXC3A and AXC4A in first 6 h. A relatively slower release of the AMK from the AXC2A film is understandable due to the higher degree swelling/expansion of AXCA3 and AXCA4, as discussed above (in expansion studies). These findings indicate that AX-CMC films extend the release time of antibiotics from the AX-based films.

To investigate the mechanism of drug release, mathematical models were fitted to release data [53]. The results (Table 3) revealed that the Korsmeyer-Peppas model fit the best, with diffusion coefficient (*n*) values of 0.5248, 0.4771, and 0.4642 for AXC2A, AXC3A, and AXC4A, respectively. These values suggest that the anomalous (non-Fickian) mechanism prevails in these formulations, as Korsmeyer et al. [49] described.

This release study concludes that the AMK-loaded AX-CMC films reported here are suitable for application in dressings for infected wounds. The initial rapid release (for 4 h) of the antibiotic could provide a rapid onset of antibacterial effects followed by a sustained effect for 24 h to treat latent infections [11]. In addition, the extended-release of the antibiotic from these films can reduce the dressing application frequency and improve patient comfort and compliance [11,12].

### 3.4. Antimicrobial Activity

The results of the in vitro antibacterial activity of the AMK-loaded AX-CMC composite films (AXC2A, AXC3A, AXC4A), blank AX-CMC film, and AMK standard solution are presented in Table 4 and depicted in Figure S5. These results illustrate that the diameter of the inhibition zones produced by the AXC2A, AXC3A, and AXC4A films (~7 mm diameter) was significantly higher (*p* < 0.05), than that of the blank film disks and blank filter paper against the tested strains. However, the zone diameters of the standard (AMK solution) and AMK-loaded films were similar, indicating that the antibacterial activity of the drug remained unaffected during the formulation process. Comparatively, the inhibition zone diameter of the AX3A film appeared to be smaller than that of the other two AMK-loaded films, although this difference was not statistically significant. Previously, AMK-loaded chitosan-based sponges (designed for local delivery) exhibited a similar antibacterial effect against *S. aureus* [25,26,28].

### 3.5. Cell Viability Assay of AX-CMC Composite Films

**Figure 6:**
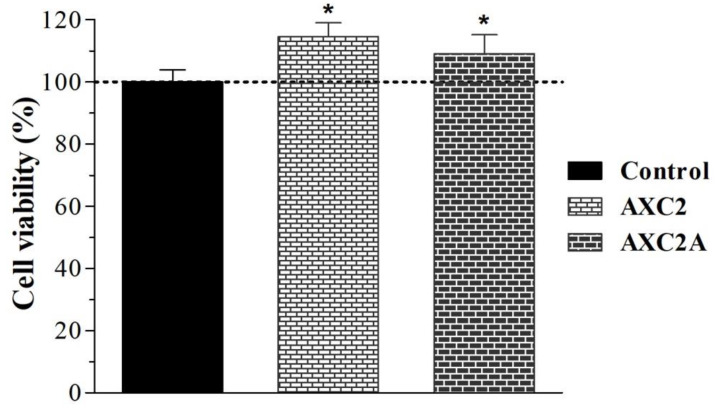
Cell viability (%) after treatment with AX-CMC films (mean ± SD, *n* = 3, * *p* < 0.05).

The cell viability test on wound dressings was carried out to assess the safety of the materials for biomedical applications [54]. For this purpose, the MTT assay was performed using MRC-5 cells. The test was performed by an indirect contact method [15] using the AXC2 and AXCA2 films. The results are shown in Figure 6. These results demonstrate that the CV (%) of the cells treated with blank and AMK-loaded AX-CMC films was significantly (*p* < 0.05) higher than that of the control cells (untreated) after 48 h of incubation. However, this value for the cells treated with AMK-loaded films (AXC2) was slightly lower than that of the blank (AXC2) film. These results indicate that the composite films under investigation are cytocompatible and the loaded AMK dose (0.2%) was not toxic to the normal cells. Hence, AMK loaded AX-CMC films can be considered safe for biomedical applications.

## 4. Conclusions

This study demonstrates that the optimized AX-CMC composite films reported here possess the desired characteristics for use in wound dressings, including swellability, flexibility, strength, cytocompatibility, and extended-release profile. The films were characterized by FTIR, XRD, and thermal analyses and found to be chemically compatible and thermally stable. The AX-CMC films were flexible and strong enough (mechanically) for application to wound sites on different body areas. The AX-CMC films can swell in simulated wound fluid (without degrading) and allow water (WVPR) exchange without dehydrating the wound. The AMK content distribution was excellent. The AMK-loaded films exhibited an initial rapid release of AMK for 4 h and a subsequent sustained release for 24 h, and in vitro tests demonstrated a remarkable antibacterial effect against the bacteria commonly found in wound infections. Thus, this study has presented a new biocompatible, biodegradable, and sustained release system based on natural biomaterials for drug delivery to an infected wound site. These films are potential candidates for preclinical studies on animal models.

## Figures and Tables

**Table 1:** Composition and physical characteristics of the AMK-loaded AX-CMC composite films.

Film Code	Composition (% *w*/*w*)				Physical Characteristics		
	AX	CMC	GLY	AMK	Peelability	Foldability	Transparency
AXC1	2	2	1	-	✓	✘	✓
AXC2	2	2	2	-	✓	✓	✓
AXC3	2	1.5	2	-	✓	✓	✓
AXC4	1.5	2	2	-	✓	✓	✓
AXC5	1.5	1.5	2	-	✘	✘	✓
AXC2A	2	2	2	0.2	✓	✓	✓
AXC3A	2	1.5	2	0.2	✓	✓	✓
AXC4A	1.5	2	2	0.2	✓	✓	✓

✓ represents Good while ✘ Not Good.

**Table 2:** Thickness, water loss, mechanical strength and water vapor permeation rate (WVPR) of the AX-CMC composite films (mean ± SD, *n* = 3).

Film	Thickness (µm)	Water loss (%)	WVPR (g m^−2^ day^−1^)	Tensile Strength (MPa)	EB (%)
AXC2	147 ± 4.1	94.9 ± 1.8	1157 ± 79	2.98 ± 0.21	118 ± 5.3
AXC3	146 ± 4.2	95.1 ± 1.2	1441± 94 ^#^	2.52 ± 0.14 ^‡^	97 ± 6.1 ^‡^
AXC4	147 ± 5.3	93.7 ± 1.6	1424 ± 74 ^#^	2.93 ± 0.12	117 ± 5.6
AXC2A	158 ± 4.5 *	93.7 ± 1.2	1142 ± 89	3.41 ± 0.13	114 ± 4.7
AXC3A	156 ± 3.7 *	94.5 ± 1.5	1495 ± 71 ^#^	2.57 ± 0.15 ^‡^	93 ± 4.5 ^‡^
AXC4A	158 ± 4.5 *	94.4 ± 1.1	1489 ± 91 ^#^	3.00 ± 0.13	113 ± 3.7

*, ^#^ and ^‡^ signs represent significant (*p* < 0.05) differences from the blank AX-CMC films, AXC2, AXC2A, and other films in the group, respectively.

**Table 3:** Amikacin release kinetics from AX-CMC composite films.

Mathematical Model		AXC2A	AXC3A	AXC4A
Zero-order	R^2^	0.8229	0.7641	0.3866
	K_o_	2.9836	2.7831	2.6271
First-order	R^2^	0.9611	0.9557	0.9611
	K_1_	−0.0382	−0.0516	−0.0380
Higuchi	R^2^	0.9535	0.9182	0.8666
	K_H_	18.046	17.143	16.612
Korsmeyer-Peppas	R^2^	0.9850	0.9853	0.9821
	K_KP_	1.3861	1.5177	1.5634
	n	0.5248	0.4771	0.4642
Hixson-Crowell	R^2^	0.8229	0.7641	0.6873
	K_HC_	−0.9945	−0.9277	0.8776

**Table 4:** Antibacterial activities of AX-CMC films (mean ± SD, *n* = 3).

Code	*S. aureus* (mm)	*E. coli* (mm)	*P. aeruginosa* (mm)
Blank Disk	9.1 ± 3.8	6.8 ± 0.1	6.8 ± 0.1
Blank Film	7.7 ± 0.5	7.1 ± 0.3	6.99 ± 0.2
AXC2A	32.9 ± 3.2 *	31.3 ± 3.1 *	29.4 ± 3.4 *
AXC3A	27.2 ± 4.7 *	28.8 ± 2.8 *	25.1 ± 2.9 *
AXC4A	31.5 ± 3.9 *	30.2 ± 4.1 *	29.3 ± 3.7 *
AMK Std.	29.9 ± 4.3 *	30.1 ± 3.9 *	30.2 ± 2.7 *

* indicate statistically significant difference from blank AX-CMC film.

## Data Availability

Not applicable.

## Supplementary Materials

The following supporting information can be downloaded at: https://www.mdpi.com/article/10.3390/polym14091769/s1, Figure S1: Optical images of Black AX-CMC Films; Figure S2: Optical images of AMK loaded AX-CMC Films; Table S1: Major thermal degradation steps of the components and selected AX-CMC films; Figure S3: TG and DSC curve of glycerol (GLY); Figure S4: Optical images of the AX-CMC composite films at different time intervals during expansion experiment; Figure S5: Inhibition diameters of (a) Blank filter disk, (b) AX-CMC blank film, (c) AXC2A films, (d) AXC3A, (e) AXC4A and (f) AMK standard.

## References

[1] Sharma A., Puri V., Kumar P., Singh I. (2021). Rifampicin-loaded alginate-gelatin fibers incorporated within transdermal films as a fiber-in-film system for wound healing applications. Membranes.

[2] Michalska-Sionkowska M., Warżyńska O., Kaczmarek-Szczepańska B., Łukowicz K., Osyczka A.M., Walczak M. (2021). Characterization of Collagen/Beta Glucan Hydrogels Crosslinked with Tannic Acid. Polymers.

[3] Michalska-Sionkowska M., Kaczmarek B., Walczak M., Sionkowska A. (2018). Antimicrobial activity of new materials based on the blends of collagen/chitosan/hyaluronic acid with gentamicin sulfate addition. Mater. Sci. Eng. C.

[4] Bialik-Wąs K., Pluta K., Malina D., Barczewski M., Malarz K., Mrozek-Wilczkiewicz A. (2021). Advanced SA/PVA-based hydrogel matrices with prolonged release of Aloe vera as promising wound dressings. Mater. Sci. Eng. C.

[5] Kanikireddy V., Varaprasad K., Jayaramudu T., Karthikeyan C., Sadiku R. (2020). Carboxymethyl cellulose-based materials for infection control and wound healing: A review. Int. J. Biol. Macromol..

[6] Jantrawut P., Bunrueangtha J., Suerthong J., Kantrong N. (2019). Fabrication and characterization of low methoxyl pectin/gelatin/carboxymethyl cellulose absorbent hydrogel film for wound dressing applications. Materials.

[7] Kurczewska J., Sawicka P., Ratajczak M., Gajęcka M., Schroeder G. (2015). Will the use of double barrier result in sustained release of vancomycin? Optimization of parameters for preparation of a new antibacterial alginate-based modern dressing. Int. J. Pharm..

[8] Maver T., Hribernik S., Mohan T., Smrke D.M., Maver U., Stana-Kleinschek K. (2015). Functional wound dressing materials with highly tunable drug release properties. RSC Adv..

[9] Basu P., Narendrakumar U., Arunachalam R., Devi S., Manjubala I. (2018). Characterization and evaluation of carboxymethyl cellulose-based films for healing of full-thickness wounds in normal and diabetic rats. ACS Omega.

[10] Kalaycıoğlu Z., Kahya N., Adımcılar V., Kaygusuz H., Torlak E., Akın-Evingür G., Erim F.B. (2020). Antibacterial nano cerium oxide/chitosan/cellulose acetate composite films as potential wound dressing. Eur. Polym. J..

[11] Alavi T., Rezvanian M., Ahmad N., Mohamad N., Ng S.-F. (2019). Pluronic-F127 composite film loaded with erythromycin for wound application: Formulation, physicomechanical and in vitro evaluations. Drug Deliv. Transl. Res..

[12] Ahmad N., Ahmad M.M., Alruwaili N.K., Alrowaili Z.A., Alomar F.A., Akhtar S., Alsaidan O.A., Alhakamy N.A., Zafar A., Elmowafy M. (2021). Antibiotic-Loaded Psyllium Husk Hemicellulose and Gelatin-Based Polymeric Films for Wound Dressing Application. Pharmaceutics.

[13] Ahmad N., Tayyeb D., Ali I., Alruwaili N.K., Ahmad W., ur Rehman A., Khan A.H., Iqbal M.S. (2020). Development and Characterization of Hemicellulose-Based Films for Antibacterial Wound-Dressing Application. Polymers.

[14] Simões D., Miguel S.P., Ribeiro M.P., Coutinho P., Mendonça A.G., Correia I.J. (2018). Recent advances on antimicrobial wound dressing: A review. Eur. J. Pharm. Biopharm..

[15] Rezvanian M., Ahmad N., Mohd Amin M.C.I., Ng S.-F. (2017). Optimization, characterization, and in vitro assessment of alginate-pectin ionic cross-linked hydrogel film for wound dressing applications. Int. J. Biol. Macromol..

[16] Hubner P., Donati N., Quines L.K.d.M., Tessaro I.C., Marcilio N.R. (2020). Gelatin-based films containing clinoptilolite-Ag for application as wound dressing. Mater. Sci. Eng. C.

[17] Alzarea A.I., Alruwaili N.K., Ahmad M.M., Munir M.U., Butt A.M., Alrowaili Z.A., Shahari M.S.B., Almalki Z.S., Alqahtani S.S., Dolzhenko A.V. (2022). Development and Characterization of Gentamicin-Loaded Arabinoxylan-Sodium Alginate Films as Antibacterial Wound Dressing. Int. J. Mol. Sci..

[18] Javanbakht S., Shaabani A. (2019). Carboxymethyl cellulose-based oral delivery systems. Int. J. Biol. Macromol..

[19] Koneru A., Dharmalingam K., Anandalakshmi R. (2020). Cellulose based nanocomposite hydrogel films consisting of sodium carboxymethylcellulose–grapefruit seed extract nanoparticles for potential wound healing applications. Int. J. Biol. Macromol..

[20] Abdollahi M., Damirchi S., Shafafi M., Rezaei M., Ariaii P. (2019). Carboxymethyl cellulose-agar biocomposite film activated with summer savory essential oil as an antimicrobial agent. Int. J. Biol. Macromol..

[21] Rezvanian M., Amin M.C.I.M., Ng S.-F. (2016). Development and physicochemical characterization of alginate composite film loaded with simvastatin as a potential wound dressing. Carbohydr. Polym..

[22] Savencu I., Iurian S., Porfire A., Bogdan C., Tomuță I. (2021). Review of advances in polymeric wound dressing films. React. Funct. Polym..

[23] Fernandes C., Acharya P.C., Bhatt S. (2018). Preparation of lauroyl grafted alginate-psyllium husk gel composite film with enhanced physicochemical, mechanical and antimicrobial properties. Sci. Rep..

[24] Maxwell A., Ghate V., Aranjani J., Lewis S. (2021). Breaking the barriers for the delivery of amikacin: Challenges, strategies, and opportunities. Life Sci..

[25] Noel S.P., Courtney H.S., Bumgardner J.D., Haggard W.O. (2010). Chitosan sponges to locally deliver amikacin and vancomycin: A pilot in vitro evaluation. Clin. Orthop. Relat. Res..

[26] Boles L.R., Awais R., Beenken K.E., Smeltzer M.S., Haggard W.O., Jessica A.J. (2018). Local delivery of amikacin and vancomycin from chitosan sponges prevent polymicrobial implant-associated biofilm. Mil. Med..

[27] Hu C., Zhang F., Long L., Kong Q., Luo R., Wang Y. (2020). Dual-responsive injectable hydrogels encapsulating drug-loaded micelles for on-demand antimicrobial activity and accelerated wound healing. J. Control. Release.

[28] Abbasi A.R., Sohail M., Minhas M.U., Khaliq T., Kousar M., Khan S., Hussain Z., Munir A. (2020). Bioinspired sodium alginate based thermosensitive hydrogel membranes for accelerated wound healing. Int. J. Biol. Macromol..

[29] Saghir S., Iqbal M.S., Koschella A., Heinze T. (2009). Ethylation of arabinoxylan from Ispaghula (*Plantago ovata*) seed husk. Carbohydr. Polym..

[30] (2018). Standard Test Method for Tensile Properties of Thin Plastic Sheeting.

[31] Surya T.G., Gurupadayya B., Venkata S.K. (2018). Spectrophotometric method for the determination of amikacin in pure and pharmaceutical dosage form. Int. J. Curr. Pharm. Res..

[32] Riss T.L., Moravec R.A., Niles A.L., Duellman S., Benink H.A., Worzella T.J., Minor L. (2016). Cell viability assays. Assay Guidance Manual [Internet].

[33] Trevisol T., Fritz A., de Souza S., Bierhalz A., Valle J. (2019). Alginate and carboxymethyl cellulose in monolayer and bilayer films as wound dressings: Effect of the polymer ratio. J. Appl. Polym. Sci..

[34] Bierhalz A.C., Moraes Â.M. (2016). Tuning the properties of alginate—Chitosan membranes by varying the viscosity and the proportions of polymers. J. Appl. Polym. Sci..

[35] Donnadio A., Ambrogi V., Pietrella D., Pica M., Sorrentino G., Casciola M. (2016). Carboxymethylcellulose films containing chlorhexidine–zirconium phosphate nanoparticles: Antibiofilm activity and cytotoxicity. RSC Adv..

[36] Thomas D., Nath M.S., Mathew N., Reshmy R., Philip E., Latha M. (2020). Alginate film modified with aloevera gel and cellulose nanocrystals for wound dressing application: Preparation, characterization and in vitro evaluation. J. Drug Deliv. Sci. Technol..

[37] Antonova N., Babichev A., Berezovsky V. (2017). Study of the morphology and structure of porous composites obtained from Na–CMC suspensions with aluminum micro-particles and boehmite nanoparticles. J. Surf. Investig. X-Ray Synchrotron Neutron Tech..

[38] Guidi A.C., de Paula M.N., Mosela M., Delanora L.A., Soares G.C.A., de Morais G.R., de Medeiros D.C., Junior A.G.d.O., Novello C.R., Baesso M.L. (2020). Stem bark extract of Poincianella pluviosa incorporated in polymer film: Evaluation of wound healing and anti-staphylococcal activities. Injury.

[39] Taheri P., Jahanmardi R., Koosha M., Abdi S. (2020). Physical, mechanical and wound healing properties of chitosan/gelatin blend films containing tannic acid and/or bacterial nanocellulose. Int. J. Biol. Macromol..

[40] Sakthiguru N., Sithique M.A. (2020). Fabrication of bioinspired chitosan/gelatin/allantoin biocomposite film for wound dressing application. Int. J. Biol. Macromol..

[41] Zhou P., Eid M., Xiong W., Ren C., Ai T., Deng Z., Li J., Li B. (2020). Comparative study between cold and hot water extracted polysaccharides from Plantago ovata seed husk by using rheological methods. Food Hydrocoll..

[42] Saadiah M.A., Zhang D., Nagao Y., Muzakir S.K., Samsudin A.S. (2019). Reducing crystallinity on thin film based CMC/PVA hybrid polymer for application as a host in polymer electrolytes. J. Non-Cryst. Solids.

[43] Kaur A., Kumar R. (2019). Enhanced bactericidal efficacy of polymer stabilized silver nanoparticles in conjugation with different classes of antibiotics. RSC Adv..

[44] Salama A. (2018). Preparation of CMC-g-P(SPMA) super adsorbent hydrogels: Exploring their capacity for MB removal from waste water. Int. J. Biol. Macromol..

[45] Bajpai S.K., Chand N., Ahuja S. (2015). Investigation of curcumin release from chitosan/cellulose micro crystals (CMC) antimicrobial films. Int. J. Biol. Macromol..

[46] Sood S., Gupta V.K., Agarwal S., Dev K., Pathania D. (2017). Controlled release of antibiotic amoxicillin drug using carboxymethyl cellulose-cl-poly(lactic acid-co-itaconic acid) hydrogel. Int. J. Biol. Macromol..

[47] Sharma U.K., Verma A., Prajapati S.K., Pandey H., Pandey A.C. (2015). In vitro, in vivo and pharmacokinetic assessment of amikacin sulphate laden polymeric nanoparticles meant for controlled ocular drug delivery. Appl. Nanosci..

[48] Malik N.S., Ahmad M., Minhas M.U. (2017). Cross-linked β-cyclodextrin and carboxymethyl cellulose hydrogels for controlled drug delivery of acyclovir. PLoS ONE.

[49] Varshosaz J., Ghaffari S., Khoshayand M.R., Atyabi F., Dehkordi A.J., Kobarfard F. (2012). Optimization of freeze-drying condition of amikacin solid lipid nanoparticles using D-optimal experimental design. Pharm. Dev. Technol..

[50] Bajpai S., Daheriya P., Ahuja S., Gupta K. (2016). Water absorption and antimicrobial behavior of physically cross linked poly (vinyl alcohol)/carrageenan films loaded with minocycline. Des. Monomers Polym..

[51] Matthews K.H., Stevens H.N.E., Auffret A.D., Humphrey M.J., Eccleston G.M. (2005). Lyophilised wafers as a drug delivery system for wound healing containing methylcellulose as a viscosity modifier. Int. J. Pharm..

[52] Vinklárková L., Masteiková R., Vetchý D., Doležel P., Bernatonienė J. (2015). Formulation of Novel Layered Sodium Carboxymethylcellulose Film Wound Dressings with Ibuprofen for Alleviating Wound Pain. BioMed. Res. Int..

[53] Setapa A., Ahmad N., Mahali S.M., Amin M.C.I.M. (2020). Mathematical Model for Estimating Parameters of Swelling Drug Delivery Devices in a Two-Phase Release. Polymers.

[54] Zhang F., Yang H., Yang Y., Wang H., Li X., Wu X. (2021). Stretchable and biocompatible bovine serum albumin fibrous films supported silver for accelerated bacteria-infected wound healing. Chem. Eng. J..

