# Supplementary material for: Arabinoxylan-Carboxymethylcellulose Composite Films for Antibiotic Delivery to Infected Wounds
Source: Polymers (Basel). 2022 Apr 27;14(9):1769. doi: 10.3390/polym14091769 (PMC9103158; doi:10.3390/polym14091769)
Supplement: Supplementary file 1 [file polymers-14-01769-s001.zip › polymers-1658313-supplementary.pdf]

Supplementary Material

Arabinoxylan-Carboxymethylcellulose Composite Films for Antibiotic Delivery to Infected Wounds

Nabil K. Alruwaili [1], Naveed Ahmad [1,*], Abdulaziz I. Alzarea [2], Fadhel A. Alomar [3], Ali Alqurain [4], Sultan Akhtar [5], Muhammad Syafiq Bin Shahari [6], Ameeduzzafar Zafar [1], Mohammed Elmowafy [1], Mohammed H. Elkomy [1], Anton V. Dolzhenko [6] and Mohammad Saeed Iqbal [7]

1 Department of Pharmaceutics, College of Pharmacy, Jouf University, Sakaka 72388, Saudi Arabia; nkalruwaili@ju.edu.sa (N.K.A.); azafar@ju.edu.sa (A.Z.); melmowafy@ju.edu.sa (M.E.); mhalkomy@ju.edu.sa (M.H.E.)

2 Department of Clinical Pharmacy, College of Pharmacy, Jouf University, Sakaka 72388, Saudi Arabia; aizarea@ju.edu.sa

3 Department of Pharmacology, College of Clinical Pharmacy, Imam Abdulrahman bin Faisal University, Dammam 31441, Saudi Arabia; falomar@iau.edu.sa

4 Department of Pharmaceutical Chemistry, Faculty of Clinical Pharmacy, Al Baha University, Al Baha 65779, Saudi Arabia; aalquraini@bu.edu.sa

5 Department of Biophysics Research, Institute for Research and Medical Consultations, Imam Abdulrahman Bin Faisal University, Dammam 31441, Saudi Arabia; suakhtar@iau.edu.sa

6 School of Pharmacy, Monash University Malaysia, Jalan Lagoon Selatan, Bandar Sunway 47500, Malaysia; muhammad.binshahari@monash.edu (M.S.B.S.); anton.dolzhenko@monash.edu (A.V.D.)

7 Department of Chemistry, Forman Christian College, Ferozepur Road, Lahore 54600, Pakistan; saeediqbal@fccollege.edu.pk

* Correspondence: nakahmad@ju.edu.sa

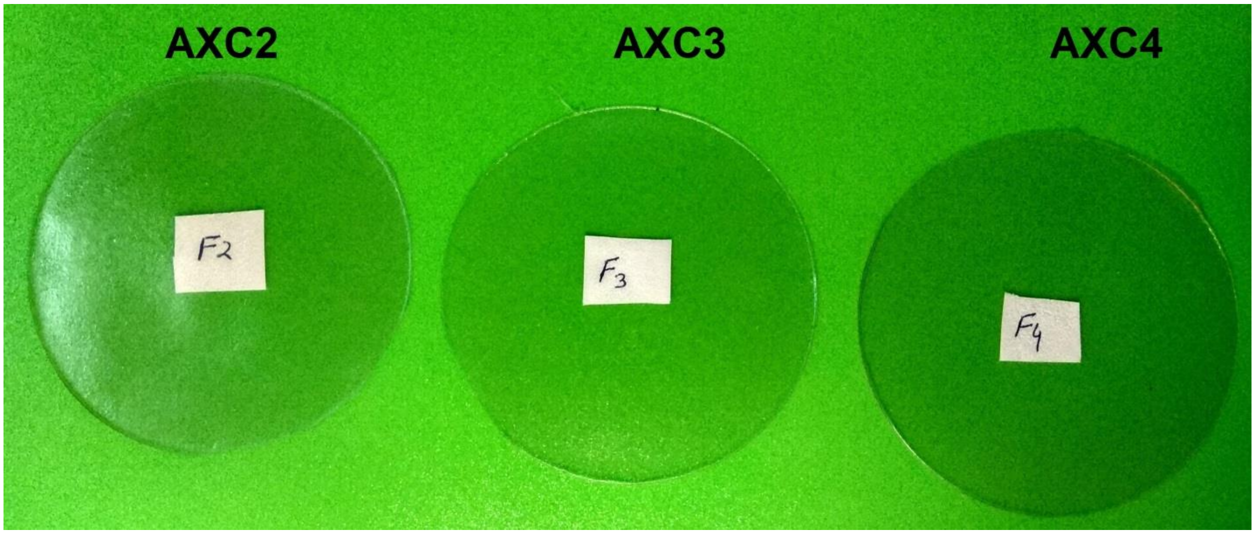

Figure S1. Optical images of Black AX-CMC Films.

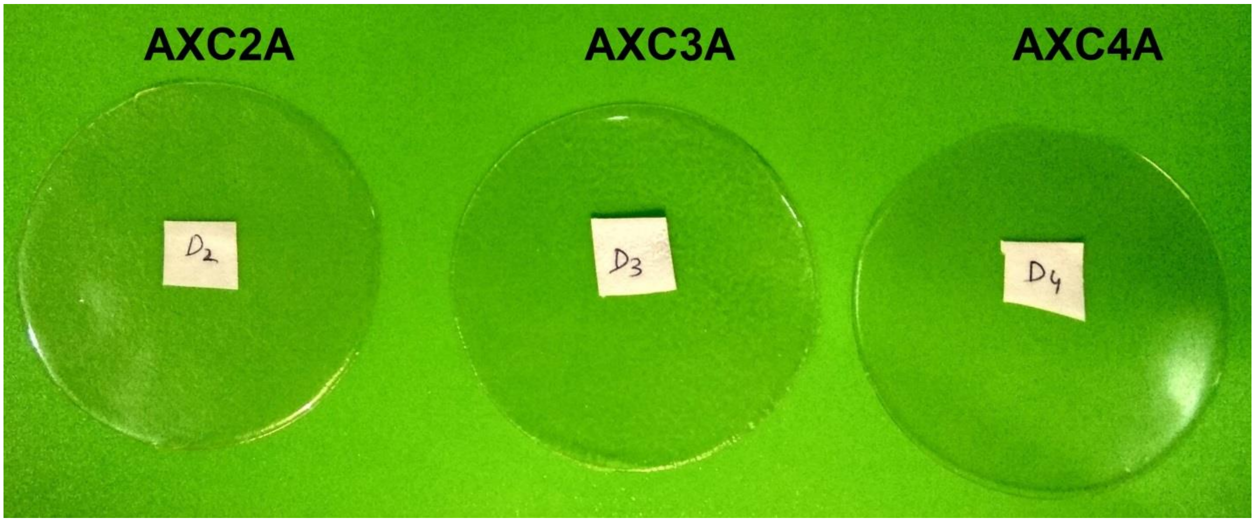

Figure S2. Optical images of AMK loaded AX-CMC Films.

Table S1. Major thermal degradation steps of the components and selected AX-CMC films.

Sample Code	First Step		Second Step		Third Step		Residue
	T_{range} (°C)	Wt. Loss (%)	T_{range} (°C)	Wt. Loss (%)	T_{range} (°C)	Wt. Loss (%)	Wt. (%)
**AX**	<125	5.27	251 to 384	42.7	>385	22.2	26.4
**CMC**	<125	5.06	256 to 335	36.6	>335	8.9	45.5
**AMK**	<130	6.26	251 to 363	31.3	>364	32.6	17.1
**GLY**	125–280	99.53	-	-	-	-	0.0
**AXC2**	<115	8.53	126 to 363	62.9	>363	7.2	22.3
**AXC2A**	<115	12.46	126 to 360	55.4	>360	7.1	24.6

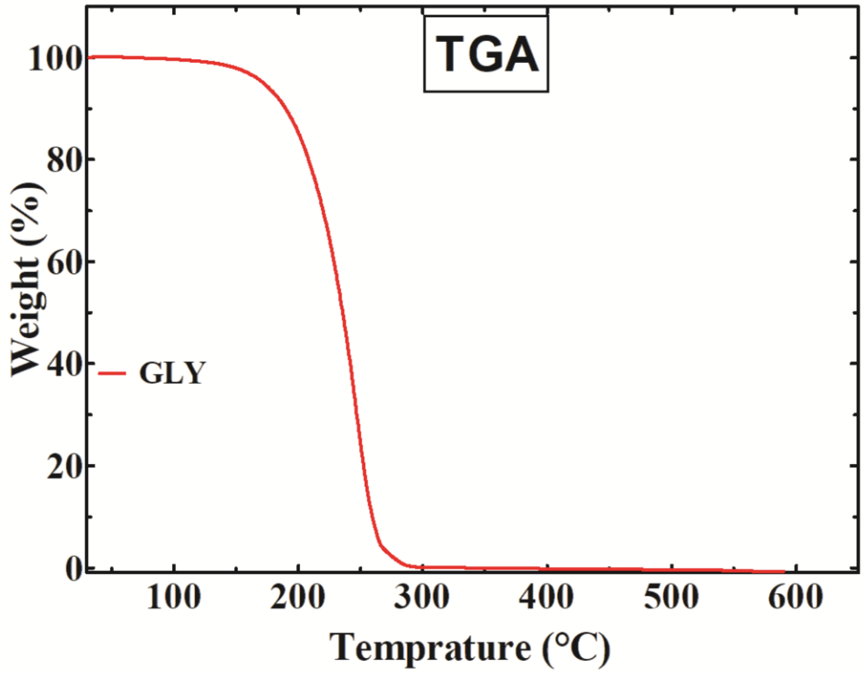

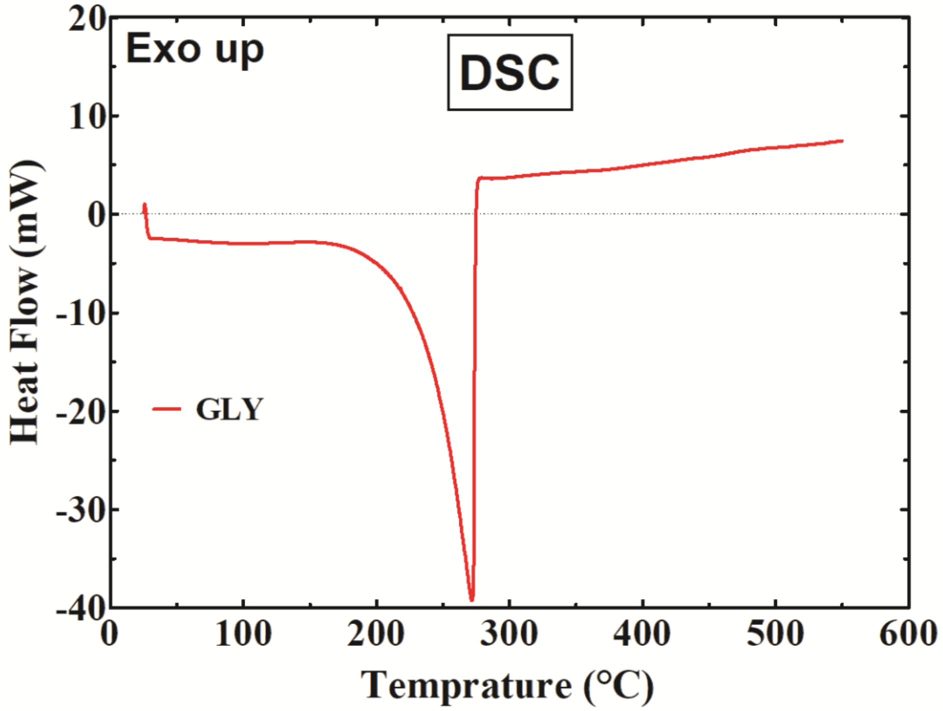

Figure S3. TG and DSC curve of glycerol (GLY).

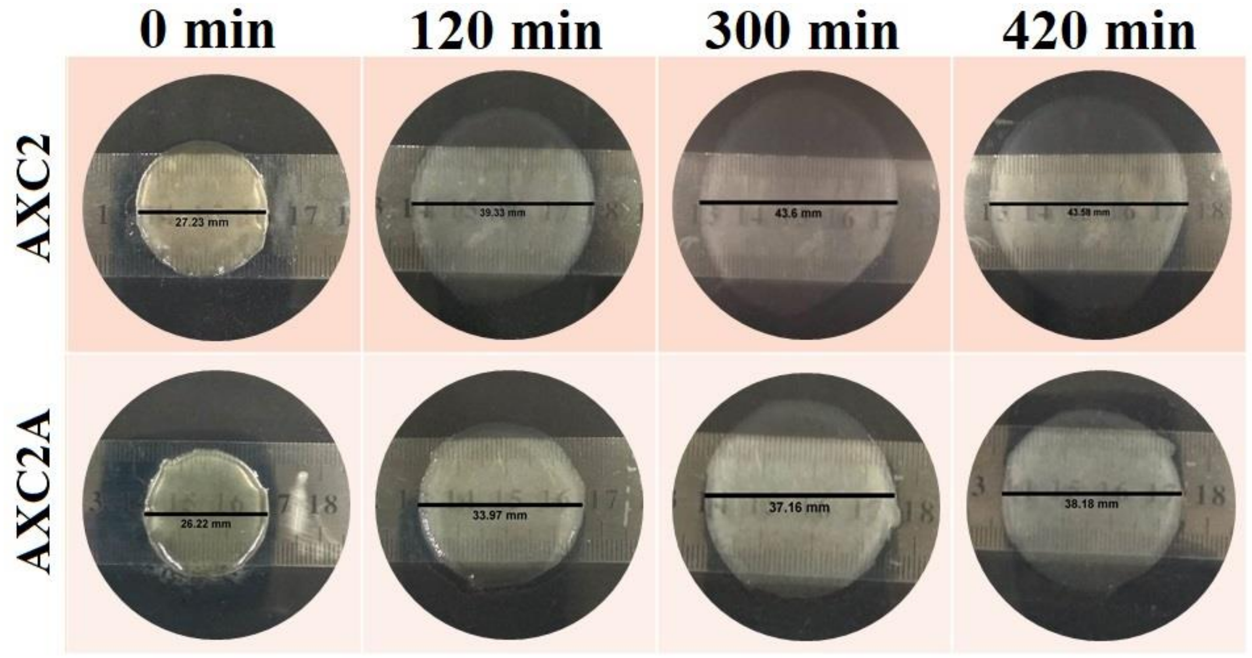

Figure S4. Optical images of the AX-CMC composite films at different time intervals during expansion experiment.

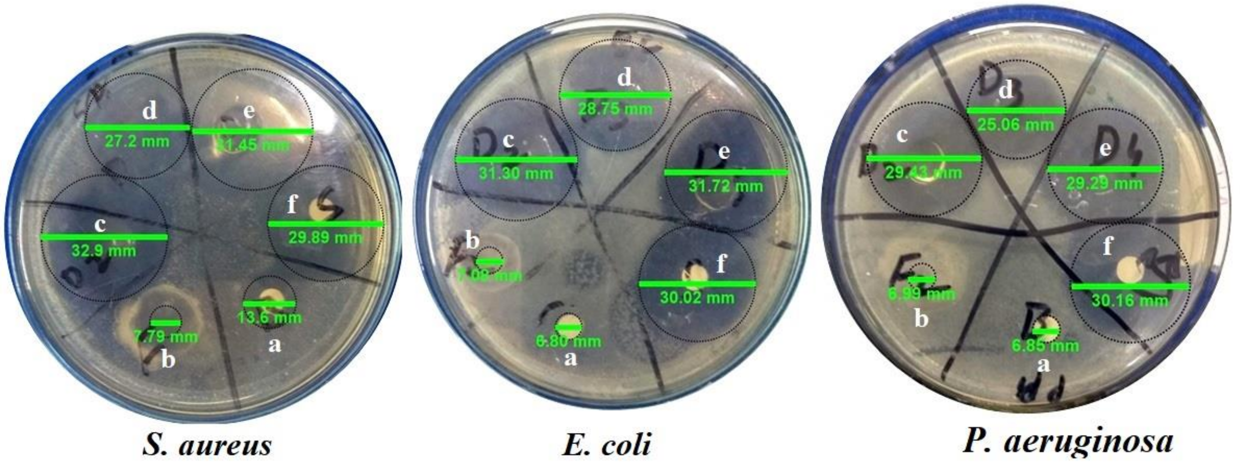

Figure S5. Inhibition diameters of (a) Blank filter disk, (b) AX-CMC blank film, (c) AXC2A films, (d) AXC3A, (e) AXC4A and (f) AMK standard.
